# Assessment of women’s needs, wishes and preferences regarding interprofessional guidance on nutrition in pregnancy – a qualitative study

**DOI:** 10.1186/s12884-024-06351-z

**Published:** 2024-02-21

**Authors:** Merle Ebinghaus, Caroline Johanna Agricola, Janne Schmittinger, Nataliya Makarova, Birgit-Christiane Zyriax

**Affiliations:** Midwifery Science-Health Care Research and Prevention, Institute for Health Services Research in Dermatology and Nursing (IVDP), University Medical Center Hamburg-Eppendorf (UKE), Martinistraße 52, 20246 Hamburg, Germany

**Keywords:** Nutrition counselling, Diet, Needs assessment, Health education, Interprofessional collaboration, Prenatal care, Midwifery care, Social media

## Abstract

**Background:**

A healthy nutrition in pregnancy supports maternal health and fetal development, decreasing the risk for adverse pregnancy outcomes. Guidance by prenatal care professionals can increase women’s awareness regarding the importance of nutrition in pregnancy and thereby contribute to a reduced risk for adverse pregnancy outcomes. The aim of this study was to assess the needs, wishes and preferences of pregnant women regarding the interprofessional guidance on nutrition in pregnancy.

**Methods:**

Using a qualitative approach and a purposive maximum variation sampling strategy, 25 pregnant women were recruited to participate in six semi-structured, guideline-oriented online focus groups. In addition, two semi-structured, guideline-oriented interviews, with a midwife and an obstetrician, were conducted. The focus groups and interviews were audio-recorded and transcribed. Transcripts were analysed using a systematic deductive-inductive approach to qualitative content analysis according to Kuckartz.

**Results:**

Focus group participants covered diverse perspectives in terms of their age, different models of prenatal care as well as dietary forms from omnivorous to vegan. The majority of women perceived the guidance on nutrition during pregnancy as insufficient. Involved healthcare professionals, namely midwives and obstetricians, should provide more consistent information, especially to avoid uncertainties exacerbated by the internet and social media. There is a need for individual nutrition information regarding dietary supplements and the specifics of different dietary forms during pregnancy, such as a vegan diet. The majority of participants supported the integration of a free-of-charge professional nutrition counselling in prenatal care. Interviews with experts identified time pressure and the complexity of nutrition as a topic as the main obstacles in consultation settings. Both midwife and obstetrician emphasised the need for improved professional education on nutrition in pregnancy in their respective studies.

**Conclusion:**

Professional guidance for pregnant women on nutrition and uncertainties going along with certain forms of diet during pregnancy could alleviate the burden and overwhelming amount of web-based information. Additionally, information adapted to the needs, wishes and preferences of pregnant women would improve prenatal care through a more personalised approach. The quality of nutrition guidance in pregnancy should be improved by the implementation of this topic in the education of involved healthcare professionals.

**Supplementary Information:**

The online version contains supplementary material available at 10.1186/s12884-024-06351-z.

## Background

The course of a woman’s pregnancy significantly affects the health of mother and child short- and long-term [1]. This warrants a focus on promoting a healthy lifestyle in pregnant women and makes health literacy, nutrition and lifestyle choices important factors in pregnancy. The understanding of health information and the interaction with healthcare professionals are associated with beneficial health behaviours as well as an increase in the health status in pregnancy, making a close engagement between prenatal healthcare professionals and pregnant women essential in promoting the health of mother and child [1–3]. In Germany, pregnant women are predominantly attended by obstetricians and are entitled to midwifery care from conception until their child’s introduction to solid foods. As part of prenatal care, the German maternity guidelines state nutrition as a topic that pregnant women should specifically be educated on [4]. So far, only few studies have investigated women’s perspectives on nutrition information in pregnancy. While there are studies that examined midwives’ role in providing nutrition advice [5], and pregnant women’s opinions on the integration of nutrition counselling in prenatal care [6], there is little corresponding research from Germany. The assessment of experiences and perspectives of pregnant women in German prenatal care is important however, considering the distinctive German healthcare system. Implementing the wishes of those who are the recipients of prenatal care can therefore contribute to quality assurance and effectiveness of that system. Moreover, communicating the importance of nutrition in pregnancy and considering women’s individual needs can be an essential step towards a reduced risk of adverse pregnancy outcomes (APO). Nutrition has been established as a major determinant of maternal and child health and a poor maternal nutritional status is associated with APOs such as preterm birth (PTB), low birth weight (LBW), gestational diabetes mellitus (GDM) and hypertensive disorders [1, 7, 8]. Additionally, dietary patterns and food quality are associated with birth weight and can impact a woman’s risk to give birth to a baby either large for gestational age (LGA) or small for gestational age (SGA), which is correlated with increased child mortality and morbidity [9]. To ensure optimal growth and development of the fetus as well as to support the mother’s physiological changes throughout pregnancy, a healthy and balanced nutrition is essential and entails the ideal amount of energy, macro- and micronutrients [10]. While the fundamentals of a healthy nutrition remain the same in pregnancy as for the general population, some nutrient requirements such as folic acid, iodine and iron are increased [11, 12]. Research shows that nutrition intervention can reduce the risk of developing preeclampsia and GDM during pregnancy [11, 13]. This entails a high intake of vegetables, whole grain products, legumes, nuts and fish, while avoiding simple sugars, processed foods, trans and saturated fats [11, 13]. Preeclampsia and GDM are, among other complications, also associated with pregnancy obesity [9, 14]. The prevalence of obesity in pregnancy has increased worldwide, which is apparent in Germany as well, where one study among many international studies showed adverse clinical outcomes for mother and child due to maternal obesity [14–16]. Apart from weight gain, many pregnant women are also concerned with a multitude of nutrition-related challenges such as food aversions, maternity sickness and heartburn [10]. Food cravings are also often reported in pregnancy and commonly increase the consumption of starchy and sweet foods as well as sugary beverages, as one Canadian study found [10]. The same study from Canada reported women’s general adherence to pregnancy-specific recommendations on food taboos to avoid the ingestion of harmful substances and the risk of infection by toxoplasmosis or listeriosis [10]. However, the authors of the study also report that women did not increase their intake of foods that are rich in nutrients [10]. The possible link of food cravings to weight gain [17] as well as the adverse impact of nutrient deficiencies in pregnancy support the need for a high-quality, evidence-based and adequate counselling on nutrition as part of prenatal care in order to improve pregnancy outcomes [18]. There has been an increase worldwide as well as in Germany of women following a vegetarian or vegan diet [19]. While a purely plant-based diet is not recommended in pregnancy [19], the increasing prevalence in the general population warrants an adequate education on the part of prenatal healthcare professionals as well as counselling pregnant women regarding regular screenings for nutrient deficiencies and recommending the appropriate dietary supplements [11]. Nonetheless, all pregnant women benefit from adequate and individual nutrition counselling, irrespective of their specific form of diet [6, 20, 21]. Nutrition as an integral part of everyday life makes it a remarkable tool in the utilisation for an immediate impact on the status of one’s own health, becoming especially relevant in pregnancy. As a topic that is specifically addressed in the German maternity guidelines, the interprofessional guidance on nutrition in prenatal care remains to be evaluated in consideration of pregnant women’s needs, wishes and preferences. Consequently, the aim of this study was to assess these needs, wishes and preferences through focus group discussions with pregnant women. In addition, we aimed to identify their sources for information and consultation on these topics and to compare the perceived importance of nutrition related topics in consultation settings to the perspectives of the involved healthcare professions.

## Methods

### Study design and participants

This study followed a qualitative approach, aiming at the exploration of individual needs, wishes and preferences through focus group discussions and interviews. The use of focus groups with pregnant women was applied to establish important aspects of their prenatal care and assess their wishes, needs and preferences to inform a demand-orientated approach in prenatal care. Focus groups were supplemented by one-on-one interviews with healthcare experts to contextualise women’s statements within the healthcare provider’s professional experiences. The study was based at the University Medical Center Hamburg-Eppendorf (UKE), Germany. It was approved by the Local Psychological Ethics Committee of the UKE (LPEK-0507) and registered at Open Science Framework (OSF) on 12.09.2022 (DOI: 10.17605/OSF.IO/YP7BR). Six semi-structured, virtually held focus groups [22] with pregnant women throughout all phases of pregnancy were conducted by M.E. during the months of September and October of 2022, via the online-platform Zoom. A virtual, semi-structured one-on-one interview [22] was conducted by M.E. at the request of one pregnant participant, who did not feel comfortable in a focus group setting. Additionally, professional healthcare providers were interviewed over the phone by M.E., in semi-structured one-on-one interviews [22], in November of 2022 and in January of 2023, including a midwife and an obstetrician. Both focus groups and interviews were conducted by female research associate M.E., who, at the time of the study, held a Bachelor of Science and had previous experience in qualitative research through participation in the conduction of focus groups and content analysis in a separate research project. Field notes were made immediately after each focus group and interview. Focus group guidelines are shown in Supplement S1 and Interview guidelines in Supplement S2. Prior to study commencement, the guidelines were audited by multiple researchers experienced in qualitative research, ensuring a clear structure and comprehensibility. Participants for this study were recruited using various methods, with the aim of obtaining a heterogeneous study population through purposive selection, to increase the diversity of perspectives and receive a wide range of insights. To achieve these maximum variation goals, we recruited participants through multiple channels, consisting of social media and distribution of flyers. Women at any stage of pregnancy, who experienced their pregnancy within the German healthcare system, who were able to join a focus group online, speak German and to give informed consent, were included. Pregnant women, who worked as midwives or obstetricians themselves, were excluded. Following the focus groups, one representative of each profession was recruited for expert interviews. The term “expert” in this context was defined as a healthcare professional with the appropriate professional education completed in Germany and at least ten years of experience working in their respective field within the German healthcare system. Prior to study commencement there was no established relationship with participants, other than contact via Email for organisational aspects and further information on the conduct of the study. The participants were aware of the researcher’s (M.E.) place of work (study center) and received an introduction into the background and reasons for conducting the study (details on the introduction in Supplement S1 and S2) as well as the researcher’s credentials and professional background. This information was given at the beginning of each focus group and interview. Focus groups and interviews were audio recorded after informed consent. Data was collected until no new ideas emerged and saturation was apparent. Focus groups lasted between 40 and 60 min, the interviews for 30 (obstetrician) and 50 min (midwife), respectively. The audio files of each focus group and interview were subsequently transcribed verbatim, using the online tool “otranscribe”.

### Data management and analysis

The management and analysis of the obtained data was performed using the 2022 version of MAXQDA. All socio-demographic data, focus group and interview recordings were stored independently from personal contact information and intentionally not interlinked.

The collected data was analysed using a concept-driven combined with a data-driven approach, reflecting the systematic approach to qualitative content analysis for a deductive-inductive analysis by Kuckartz [23]. The applied framework method was described by Gale et al. [24] and entails the organisation of codes into categories. Based on the focus group guideline and the first impression after reading and summarising all transcripts, the main categories were deductively developed, reflecting the overall themes covered by the discussions. All transcripts were then coded with an inductive approach, using the emerging ideas directly from within the data to extend the main category system with more subcategories until they appeared saturated. The resulting category system was revised by multiple researchers with experience in qualitative research. After initial coding of the data by one researcher, a second researcher, with extensive experience in qualitative research, independently applied the developed category system for one focus group transcript. Aiming for agreement of coding and inter-coder consistency, existing disagreements were discussed between the researchers until consensus was reached. The final category system for the focus groups was subsequently applied to the interview transcripts. We performed translation from German to English as closely to the original as possible.

## Results

### Sample characteristics

Six focus groups and one interview were conducted with pregnant women, as well as two expert interviews with healthcare professionals. A total of *n* = 25 pregnant women participated in the study. Women were between the ages of 23 and 38 years, with a mean age of 30.9 (SD: 3.4). The week of pregnancy ranged from 8 to 40 weeks. All participants had a high school diploma (Abitur) and *n* = 13 completed college education. The participating women had chosen three different models of care for their pregnancy, either receiving prenatal care exclusively from their obstetrician (*n* = 10), in an alternating model between midwife and obstetrician (*n* = 11) or in a midwifery-led model of care (*n* = 4). Regarding their form of nutrition, participants followed a variety of diets. More than half (*n* = 14) stated that they are omnivores, *n* = 2 women indicated a pescetarian and flexitarian diet, respectively, while *n* = 5 women were vegetarians and *n* = 2 followed a vegan diet. Supplement S3 summarises the socio-demographics of the focus group participants. The second part of this study consisted of two expert interviews with healthcare professionals, a midwife and an obstetrician. The interview participants completed their professional education in Germany and had multiple years of experience in their field.

### Category system

The development of the category system followed a deductive-inductive approach [23]. The main categories are “source of information”, “topics”, “timing of consultation”, “communication between healthcare experts and pregnant women”, “offered services”, and “professional education of experts”. The final category system is shown in Supplement S4.

### Guidance on nutrition during pregnancy

#### Source of information

##### Focus groups

Women reported three main sources of information on nutrition during pregnancy. Healthcare professionals, the internet and social media, while the third source for these women were personal contacts, giving advice on nutrition in pregnancy, albeit sometimes unsolicited. Healthcare professionals who advised the women on nutrition during pregnancy were their obstetricians and midwives. Some women recounted a more comprehensive nutrition counselling by their midwives than their obstetricians, often as a result of more time in prenatal visits. Many women reported they had not received any guidance or education neither by their midwife nor by their obstetrician, which they would have wished for in retrospect. For some women this led to the feeling of not being able to rely on their healthcare professional to address the topic.

*“I don’t remember any significant information or education given by my obstetrician regarding that. And looking back, I would have appreciated it.”* (30 years, 1st child).

Interestingly, one woman stated that she does not necessarily believe healthcare professionals in pregnancy to be the most reliable source for nutrition information and found that a medical training is no guarantee for accurate information on nutrition in pregnancy.

*“The problem that I see, when it comes to nutrition, is that specifically medical professionals are not necessarily more trained than someone who deals with it a lot or otherwise works scientifically. So, unfortunately, in my experience, a medical background is not a guarantee for reliable information when it comes to nutrition.”* (28 years, 2nd child).

Some participants pointed out that the alternating model in prenatal care between midwife and obstetrician could prevent a lack of guidance, as they were not certain, they would have received such an extensive counselling if they had solely been in obstetrical care and not midwifery care as well.

*“If I imagine now that I wouldn’t have had any midwifery care and only the obstetricians, I don’t know if they would have told me about it. Or, I would have wished for it from the obstetrical side as well.”* (35 years, 1st child).

Many women saw an issue with the consistency of information they received from different healthcare professions. Women recounted inter- and intraprofessionally inconsistent information on the topic of nutrition, adding to the uncertainty they already experienced around this topic. Consequently, many women stated they needed to find their own way, recognising that they are responsible for their decisions. Many wished however for a more consistent picture and a general consensus between the involved professions.

*“So when you hear such contradictory opinions from two professionals, to know, well, which is true? I always feel like in the end, it’s my decision. I have to inform myself for myself about how far I want to go and accept the information that I want.”* (23 years, 1st child).

*“I would have wished for a more consistent picture. Everyone told me something different about nutrition.”* (35 years, 1st child).

Regarding other sources than healthcare professionals for nutrition information, a considerable amount of time in focus group discussions was occupied by the topic of social media and the internet in general. Most women stated they used the internet as a research platform, often seeking information on which foods they were allowed to eat during pregnancy. Many women explained the need for internet research with the insufficient or lack of counselling by their prenatal healthcare professionals, some stating they did not feel adequately informed.

*“Regarding nutrition, I actually received very little information and had to tediously do the research myself.”* (26 years, 1st child).

This became especially apparent regarding a lacking education on GDM, with one woman recounting how she felt when her obstetrician suspected she might be affected.

*“So, I ended up googling what would happen if* [GDM] *turned out to be true and everything. That really wasn’t good because I didn’t feel like I was well-informed.”* (30 years, 1st child).

The pervasive nature of the internet introduced a number of concerns for pregnant women, including the multitude of different opinions presented on, for example, social media channels. Some women also pointed out the additional effort to distinguish between online sources trying to sell a certain product, often dietary supplements, and sites without any financial agenda. Many women concluded the necessity to evaluate for themselves which information is useful and individually fitting depending on their situation and personal needs, describing the amount of information as overwhelming and difficult to navigate, especially in their first pregnancy. Some participants also pointed out positive aspects of the internet as a source for nutrition information, serving a quick reinsurance when in doubt about certain food taboos and finding individual solutions.

*“When it comes to nutrition, I find it extremely difficult to filter, who is trying to sell a product. Because it’s especially extreme in this pregnancy bubble, the amount of dietary supplements being sold. And what are actually reliable sources without any advertising motives.”* (28 years, 2nd child).

In response to what kind of support would be helpful in navigating the internet and social media as a source for information on nutrition, some women pointed to government official websites or apps, meaning evidence based and approved by responsible German ministries. Facilitating the search for reliable answers, specifically on food taboos, women voiced the wish for free-of-charge apps and official websites.

*“An official app or an official website would certainly create more trust.”* (33 years, 1st child).

Women also wished for written information from their healthcare professionals on specific topics such as GDM, to avoid feeling caught off guard or scared later on and knowing how to use nutrition as a tool to prevent GDM. Another wish was expressed regarding a catalogue on beneficial foods during pregnancy or a list of dietary supplements that could be helpful, together with an explanation on each supplement.

*“I would have wished for something either from my obstetrician or my midwife, like lists with nutrition pyramids or generally, what are the superfoods to eat during pregnancy, what should I eat or focus on? If I had only received some kind of informational booklet about what to eat during pregnancy or what is recommended.”* (34 years, 1st child).

Lastly, women reported personal contacts as a source of information on nutrition and stated they possess a basic knowledge through their families and friends who have experienced pregnancy. Sometimes however this led to confusion when their advice did not coincide with information from other sources.

*“Especially because you always have the mothers in the background or the grandmas, saying, oh, we ate everything, we didn’t know about all that stuff. You have both extremes. The friend who freaks out, saying how could you? Then I feel like, non-pregnant people also like to get involved. And on the other side, the moms, or aunts, grandmas.”* (32 years, 2nd child).

### Topics

#### Focus groups

Women were concerned with many different topics regarding their nutrition during pregnancy. As already apparent throughout the theme of women’s sources, an important aspect of this were foods that are labeled as a taboo during pregnancy, often in relation to a risk of infection.

*“What is much more important is all this infection stuff right at the beginning. And there, I would have wished for something a little more differentiated. A bit more detailed, having more faith in our intelligence. I can remember more than two things (laughs). It’s such a generalisation.”* (35 years, 1st child).

Other topics included women’s diets and special nutrient requirements during pregnancy. Participants also addressed nutrition-related health matters and the health of their unborn infant. Many women talked about trying to maintain a healthy nutrition during pregnancy and about what that entails.

*“Especially in pregnancy, you want to somehow get the most out of it for yourself and the baby, making sure to have all the necessary vitamins and minerals and not forget anything, that everything is included in your nutrition.”* (26 years, 1st child).

Pregnant women who received a more extensive education on nutrition reported a positive impact on their ability to evaluate what a healthy nutrition actually means.

*“I think nutrition is something that concerns all of us pregnant women and we want to eat healthily. But what does healthy actually mean and what is / how can you determine this? I really liked that. I think it was great that* [the midwives] *implemented this.”* (35 years, 1st child).

An important issue for many women was the continuation of their previous specific form of diet, such as vegan, vegetarian or omnivorous diet, and the impact this has in their pregnancy. Many women voiced their expectation and wish to be asked by their prenatal healthcare professionals about their specific diet and were disappointed or confused, when neither their midwife nor their obstetrician inquired any further. This was especially addressed by women who followed a vegetarian or vegan diet, or who had special restrictions, such as a gluten free diet. These women wished for a more individual consultation by their midwife or obstetrician regarding particular requirements these diets entail.

*“I was also surprised that during my first appointment with the obstetrician, he didn’t ask about my diet. And I found that very strange. He just said something about being careful with meat and cheese. But at the time I thought, well, that doesn’t really apply to me, but I just let him talk.”* (32 years, 1st child).

Often in relation to a vegetarian or vegan diet but also voiced by many other participants was the topic of nutrient requirements specific for pregnancy. While many were educated on the importance of folic acid at the beginning of their pregnancy, women voiced a specific need to be informed on more nutrients and in more detail.

*“I would have wished for a list of what I can take, and it should be optional. Maybe during another appointment with my obstetrician or maybe as part of the prenatal care with the midwife. Does it make sense for me? Should I take it? So, maybe a bit more individualised.”* (32 years, 2nd child).

During focus group discussions, some women reported pregnancy-related issues such as maternity sickness, heartburn or insatiable appetite. A great concern for many was also GDM. For many participants, a suspected GDM was reason for them to inquire more about the impact of nutrition. In this context, they expressed the need to be educated earlier in their pregnancy on the preventive effects of nutrition by their prenatal healthcare professionals.

*“I read a lot about* [GDM], *but there was little information from* [the obstetrician] *directly. So, I would have wished for more, earlier clarification on the topic.”* (34 years, 1st child).

### Interviews

The interviews with the midwife and the obstetrician reflected the main topics related to nutrition and pregnancy, which had previously been outlined in the focus groups by the pregnant participants. Both the midwife and the obstetrician placed special focus on the risk of infection through certain foods and the harmfulness of alcohol, smoking and drugs.

*“And specifically with regard to toxoplasmosis, listeriosis, women are informed that they should not eat any raw milk products, any raw meat products, and so on.”* (Obstetrician).

The midwife and obstetrician both noted that pregnancy seems to be a great motivator for women to eat healthier and give their child the best possible starting conditions. They also stated that vegetarian and vegan diets give reason to consult on nutrition in more detail with the respective women.

*“This taking of responsibility for the child is also reflected in women’s nutrition and they don’t want to do anything wrong.”* (Midwife).

Regarding dietary supplements, the obstetrician talked about folic acid and iodine as a recommendation and any other supplements as a possibility. Interestingly, the midwife pointed out that folic acid is justifiably very important to obstetricians but in her experience, they do not convey a sufficient explanation on this to the women.

*“Folic acid is very important to obstetricians. But instead of explaining that it is difficult to reach the folic acid levels that we need during this special time through nutrition, which women often don’t know. The background information is not communicated.”* (Midwife).

Maternity sickness was briefly mentioned by both midwife and obstetrician in context of the fear many women have that they might not be eating enough to sufficiently provide the unborn child with nutrients.

*“Many women in their first pregnancy are concerned that their child may not be getting enough nutrients. They always fear that the child is missing something. And then you can show them the child and say that the child is growing just fine and it will take what it needs.”* (Obstetrician).

### Timing of consultation

#### Focus groups

Regarding the timing of nutrition education during pregnancy, many women emphasised how overwhelmed they felt early in their pregnancy by the amount of information they received. Nonetheless, they saw the need for a consultation on the topic of nutrition very early on. Many women would like nutrition to be addressed by their midwife and obstetrician on multiple occasions during their prenatal appointments, as questions might change or arise overtime and the initial feeling of being overwhelmed fades.

*“Some questions only come with time. I think it should maybe be addressed again and again, also actively brought up by midwives or obstetricians maybe. Revisited again as time goes on.”* (33 years, 2nd child).

Recurrence of the nutrition topic was a frequent point made by women who suffered from maternity sickness early in their pregnancy and could not cope with nutrition counselling at that time.

*“I also experienced during this pregnancy that I was just feeling sick for a long time and had to throw up constantly. And I can imagine that if it’s talked about during that period, it would go in one ear and out the other. Therefore, it wouldn’t be a bad idea to repeat it again later.”* (32 years, 2nd child).

Some women therefore proposed an early education on the risk of infection in the context of toxoplasmosis and listeriosis and a second consultation on nutrition and GDM in the first weeks of the second trimester, when women felt more settled in their pregnancy.

*“I believe a good time for a second consultation would be around the 12th week. I also think at that point, the initial excitement and anxiety that many women feel during the first two to three appointments in the practice would have subsided a bit. And I also believe that would be early enough to educate on gestational diabetes.”* (33 years, 1st child).

### Interviews

The dilemma of wanting to be informed but at the same time feeling overwhelmed by the amount of information women are exposed to at the beginning of their pregnancy was also acknowledged by the interviewed midwife. In her experience, nutrition as a topic has a very negative association for many women due to maternity sickness. Similar to what was described in focus groups, she saw the second trimester as the best time to offer nutrition consultation, explaining this with the women’s increasing sense of their pregnancy and awareness of carrying a child.

*“At the beginning of pregnancy, women are often confused about nutrition. Because they feel nauseous for up to nine, ten, eleven, twelve weeks and the topic of eating is pushed very far away. After that there is a switch. Then there is the feeling of being pregnant and feeling the baby move. But up until that point, women are preoccupied with many other things to adjust to the changes in their hormone levels. Therefore, discussing nutrition or reducing sugar intake and other things at this point - she can’t focus on that.”* (Midwife).

The need for written information in addition to a verbal consultation was reflected by the interviews with midwife and obstetrician, stating written material would facilitate the counselling on nutrition and alleviate the overwhelming amount of information in the beginning.

*“So these flyers really make it easier, and things to take with you. Because there is a lot of information at the first appointment, but also important. So we usually say, this is a lot of information, that’s why you get all these flyers and let it sink in first.”* (Obstetrician).

### Communication between Healthcare experts and pregnant women

#### Focus groups

An overall theme throughout the focus group discussions that was valued very highly by pregnant women was the importance of communication. Due to pregnancy being a special time in a woman’s life, the interpersonal relationship between women and their prenatal healthcare professionals was considered essential. Many focus group participants wished for a personalised consultation, taking into account their individual tendencies to get easily scared or feeling unsure, or their personal choices regarding their nutrition, and considering the ability of a woman to cope with the amount of information they are presented with on the topic of nutrition. Some women reported they felt left alone and afraid of doing something wrong, which was especially apparent in the context of food taboos. The women concluded that guidance by healthcare professionals is essential in putting risks and fears into perspective.

*“You are initially left alone with all these fears and you are afraid of everything. It’s important to take the pressure off a bit and just say, okay, what is reasonable, what is just scaremongering, and what eventually would also become unhealthy.”* (35 years, 1st child).

Some women reported they wished they had been given more guidance by their obstetrician to avoid worrying about making mistakes regarding their nutrition. Some focus groups participants even stated, they were somewhat frightened by their obstetrician’s warnings about risks of infections through foods or nutrient deficiencies, especially in vegan diets.

*“I can’t even remember my obstetrician saying anything about nutrition. And I was really anxious about the whole topic. I think it’s totally unnecessary to get so worked up about it. And in hindsight, I thought to myself, my obstetrician is my person of trust. If she had given me a little bit of a guide or something from the beginning, then I don’t think I would have gotten so worked up about some things afterwards.”* (30 years, 1st child).

### Interview

The midwife also emphasised the importance of the relationship between her and the pregnant women. In her experience, a good relationship can have a positive effect on the overall course of pregnancy and can help women to cope with fears, especially in regards to nutrition.

*“I have the impression that when women are closely bound to their midwife, they go through pregnancy with more ease and confidence, especially regarding nutrition and possible fears, questions, and concerns.”* (Midwife).

Referencing the framework conditions that midwives work in, the interviewed midwife noted the amount of time spent consulting women in prenatal visits, and the wish for the topic of nutrition to be specifically addressed in the catalogue of preventive services that midwives can bill for. She stated that topic-related billing options could improve prenatal care, as it would motivate healthcare professionals to incorporate nutrition topics in their care for pregnant women.

*“I believe that if there were better conditions for preventive care services, if there were proper billing points for them, then it would also be more focused on. If one were to further break down aspects of billing possibilities and have a topic-specific billing that is also coherent, for example, nutrition counselling or nutrition for dental health, weight counselling, if that were further broken down, then every midwife would have a proper catalogue to work through.”* (Midwife).

Time pressure was a big concern for the interviewed obstetrician, which she stated can make the counselling on nutrition difficult. She explained this with the complexity of this topic that also goes along with ingrained habits and the psychology behind trying to change them.

*“What makes educating difficult? Well, there’s time pressure, of course. That’s the case in all practices. And of course, if someone is not cognitively able, then it’s also a challenging story and you never know what they will take away from it, except for the ultrasound image (laughs). Nutrition is a specific topic that deals with habits, ingrained behaviour, and also knowledge.”* (Obstetrician).

### Offered services

#### Focus groups

In terms of nutrition-related prenatal medical services that women need or appreciate during their pregnancy, two main talking points emerged in the focus group discussions. Firstly, the topic of cost coverage of certain services by their health insurance was addressed and secondly the need for offers of consultation and information proactively provided by healthcare professionals. Especially for folic acid many women voiced their wish for cost coverage of this supplement, at least in the beginning of pregnancy, as it is a standard recommendation but rarely covered by health insurance. Moreover, some women would like for the cost-intensive multivitamin, often recommended by obstetricians, to be covered as well. Many pointed out, that not every woman has the means to cover the cost for supplements herself.

*“I also think it’s somewhat of an audacity in the German healthcare system that it’s more or less required with folic acid, the ultimate dietary supplement, and it’s not covered by health insurance. I don’t know why it’s not considered in this context to provide a financial relief for pregnant women.”* (23 years, 1st child).

One woman suggested that an education on how to obtain important nutrients via nutrition by healthcare professionals could replace a cost-intensive management with dietary supplements. Building on this theme, many women expressed their wish for a one-time free-of-charge nutrition counselling for pregnancy, especially for women in their first pregnancy.

*“I believe there are many pregnant women who can’t afford to take all the vitamins you can take, omega-3 fatty acids, and so on. And of course, it’s possible to manage this through your nutrition, and even manage it more cost-effectively through nutrition. So, there it would be good to receive an education. A pregnancy nutrition counselling service that could be offered once for free during pregnancy or for first-time mothers would be very helpful. Of course, we would have to consider who would pay for it, but it would be very, very valuable.”* (33 years, 1st child).

Furthermore, the focus group participants wished for more proactive consultation and information offers by their midwife and obstetrician regarding nutrition in general. They emphasised the need for healthcare professionals to inquire about the woman’s state of knowledge about nutrition in pregnancy, signal a willingness to help, actively address the topic during multiple prenatal visits and prepare a questionnaire to avoid missing any important information the woman might forget to mention herself.

*“I think the midwife or obstetrician or both should also ask about the level of knowledge of the pregnant women, whether they have any questions, and whether they can still help. So, the topic should be addressed by them and they should offer help. As a pregnant woman, you may not remember everything during the appointment with the midwife or obstetrician, so that they really ask about everything because it’s really, really important.”* (33 years, 1st child).

Women also wished for specific guidance on how to compose meals that are rich in nutrients, as they feel there is an expectation to eat a balanced diet, but no offers for information on how to do so. A wish for more support was also mentioned in the context of maternity sickness and the disappointment at the lack of advice on how to counteract this through nutrition.

*“I would have wished for a consultation offer because the nutrient requirements are increased and it would be helpful to have an information offer on how to put together my meals in a smart and nutritious way. Is that information accessible to everyone? Or would it be maybe smart to offer information about that?”* (28 years, 2nd child).

### Interviews

In the interviews with midwife and obstetrician some of the topics that were discussed by the pregnant participants recurred while a few new ones emerged as well. The obstetrician touched upon the issue of cost coverage by health insurance.

*“Some health insurance companies reimburse certain things, for example they pay for a toxoplasmosis test, which is not a standard benefit in prenatal care. But ultimately, patients have to pay for it themselves, and if it were a standard benefit, it could be offered to everyone. So, this is maybe something else to consider.”* (Obstetrician).

The obstetrician also stated, she would appreciate the opportunity and would be open to refer pregnant women to one free-of-charge nutrition counselling, if this were included in health insurance programs, as she recognised that it is otherwise too expensive and as a result not considered by most women.

*“Nutrition counselling is of course great, but for most people it’s simply too expensive. But if a health insurance company would include such a program, I would be very open to it. If they were to say, okay, you have the opportunity to go to one nutrition counselling session.”* (Obstetrician).

The importance but also time-consuming nature of a nutrition consultation was highlighted by the midwife, who did not have the impression that it is adequately addressed by obstetricians.

*“And these are actually very extensive conversations. And this happens within the prenatal care with every woman, and how she reacts to how this conversation* [with the obstetrician] *went and whether there is still something there, whether there is still a need for discussion.”* (Midwife).

#### Professional education

##### Interviews

The prenatal healthcare professionals, interviewed in this study, did not feel adequately prepared by their professional education on the topic of nutrition in pregnancy. They personally had obtained further information through additional training programs or their respective professional associations. The interviewees agreed on the importance of the topic and the need to receive basic nutrition knowledge through their professional education. However, as the obstetrician pointed out regarding her experience during her medical studies, nutrition in pregnancy is a specific topic mostly relevant for those specialising in obstetrics.

*“In the end, basic knowledge should of course be there. But there are many people who are not interested in it if they don’t go into obstetrics later on.”* (Obstetrician).

The midwife recognised a certain backlog regarding nutrition counselling in pregnancy, while also noting that contents of professional education, be it medical studies or midwifery training, might be different today.

*“There was a huge need to catch up on my knowledge, but I didn’t really notice it for a long time. However, during my time as a midwife, I realised that it was not enough, that there were simply topics that I was not familiar with. And I had to catch up and learn more through training and literature.”* (Midwife).

## Discussion

With this qualitative study, we sought to assess pregnant women’s needs, wishes and preferences regarding sources for nutrition information and consultation on related topics. This was complemented and contextualised with impressions of involved healthcare experts, in consideration of interprofessional prenatal care. Women wished for a personalised counselling, catering to their individual needs, determined by factors such as their individual knowledge, coping abilities, their lifestyle and specific diet. The results of this study also show women’s need for clarity and intra- as well as interprofessional consistency regarding the information they receive from healthcare professionals. Focus group participants were in some cases offered a comprehensive consultation by their midwives, while obstetricians usually covered basic and general recommendations in terms of food taboos in pregnancy. Compared to women exclusively cared for by obstetricians, women in an alternating care model received a more extensive education in terms of nutrition, due to their midwifery care. However, the lack of personalised advice on nutrition and specific forms of diet, and the inconsistency of information from healthcare professionals resulted in a self-reliant internet research, which in turn exacerbated the already existing uncertainty and anxiety surrounding this topic for pregnant women. The study results identified social media as an integral part for pregnant women’s nutrition information rather than having received a satisfactory guidance on nutrition in pregnancy by healthcare professionals. The impact of the internet and social media in information-seeking on nutrition-related issues has previously been pointed out by multiple studies, amongst others [21, 25, 26] by Aktaç et al. [20] in pregnant Turkish women and by Bianchi et al. [27] in pregnant French women, who outline the increasing usage of the internet in answering immediate health-related questions and its contribution to anxiety, due to inconsistent information. This has also been reiterated by a Swedish study, who reported that most women searched the internet for pregnancy-related information, even despite the satisfying information some received from their prenatal healthcare professionals, causing feelings of worry [28]. A study that recently reviewed leading websites offering guidance on nutrition reported that women access information via the internet that is not in compliance with evidence-based guidelines and concluded a need for updated data on those websites [29]. Further results of our study indicate that pregnant women and healthcare professionals are concerned with similar topics, show however differences in the weighting of their importance. There is an agreement on the indisputable relevance of food taboos and the risk of infection through certain foods. Especially important to pregnant women was the way healthcare professionals communicate this, and our results show that providing differentiated information and putting potential risks into perspective rather than aggravating fears in respect to nutrition can enhance women’s health literacy and feeling of competence. Pregnant women’s concerns about nutrient supply during pregnancy were shown to be important not only to them but were also recognised by healthcare professionals. However, the results indicate that the topic of dietary supplements is much more worrisome for women than prenatal healthcare professionals realise. This suggests the necessity for healthcare experts to learn more about and implement personalised advice on the significance of different nutrient requirements in women’s individual pregnancies, particularly but not exclusively for those who follow a vegetarian or vegan diet. Advising women on essential and non-essential dietary supplements might alleviate the perceived burden of women who are concerned with the health of their child and worried about the impact of nutritional deficiencies. Research shows that an educational nutrition intervention could improve women’s knowledge of nutrition [30], while the promotion of nutrition awareness by healthcare professionals has additionally been shown to be more effective and longer lasting when interactive and according to women’s interests [31]. The benefits of personalised health information have been demonstrated by multiple studies on nutrition counselling and other various outcomes, showing that it can improve the adherence to diet and lifestyle interventions [32–35]. In the context of additional costs in pregnancy, the coverage of dietary supplements by health insurances could support women financially as many participants pointed out, especially for those who already struggle financially due to pregnancy-related expenses. A multivitamin preparation containing among other micronutrients folic acid is recommended by many obstetricians to women in pregnancy and preferred over individual dietary supplements, which became apparent during focus group discussions. In Germany, some health insurance companies reimburse costs for individual supplements such as magnesium, iodine, folic acid or iron [36], however, a multivitamin preparation must be paid by the consumers themselves. The reality is, which this study also demonstrates, that due to convenience and need for reassurance of obtaining all important micronutrients, pregnant women pay for multivitamins instead of selecting individual supplements that might be reimbursed. Additionally, due to dietary supplements being regulated by food laws in Germany, the market and quality control of those products lacks transparency and makes the selection more difficult [37]. It is important however to keep in mind that the participants of this study had a relatively high educational status, which is associated with higher adherence to supplementation [38]. The women additionally had the means to cover the expenses themselves, nevertheless mentioned the burden of those costs, especially women who were still in university at the time of the focus groups. While this has not been discussed specifically for Germany, studies from other countries show that free-of-charge folic acid leads to a higher adherence to supplementation and as a result fewer APOs such as spina bifida [39]. Beyond a cost-coverage of multivitamin preparations for pregnancy, providing women with a comprehensive education on essential nutrients and dietary supplements as well as a consultation on how to include those in everyday nutrition, which was voiced as a wish by many focus group participants, could decrease women’s expenses in supplements and improve motivation to do so, as previously pointed out by Zeng et al. as well [39]. Furthermore, the results of this study point to the empowerment that women can feel through their healthcare professionals when given information and helpful sources to gain control over their nutrition and lifestyle. However, the results of our study show a lack of professional preparation in respect to the expert’s education on nutrition in pregnancy, leading to privately obtained information by healthcare professionals. Study participants recognised as problematic that this circumstance can lead to very different outcomes regarding the advice pregnant women receive, due to its dependence on the self-education of their respective healthcare professionals, as a medical education is no guarantee for adequate nutrition information [5, 6, 40]. The women interviewed in this study pointed out their self-responsibility to care for their own health, wish however for the tools to do so provided by their healthcare professionals. Our study also suggests that healthcare professionals have the ability to either empower or unsettle the women in their care, which emphasises the importance of communication. The effect their statements have on pregnant women was also recognised by the interviewed midwife who believes that midwifery care can reinforce women’s confidence and ease the fears and concerns regarding, among other topics, nutrition. Interestingly, this aspect of midwifery care has previously also been discussed by Mattern et al., demonstrating that a reassuring care by midwives can increase equanimity and self-confidence in pregnant women [41]. Taking the time and creating a calming environment has been appreciated by the participants of this study and constitutes somewhat of an advantage midwives have in their approach to prenatal care, where they are usually able to dedicate more time to individual issues such as nutrition [41] as compared to obstetricians. Research has shown an increase of nutrition awareness in pregnant women, depicting pregnancy as a life event that can trigger motivation to eat healthier and be more interested in nutrition-related information [30, 31, 42, 43], while interestingly, studies show limited knowledge of pregnant women about specific impacts of nutrition [21, 43, 44]. Finding relevant material and reliable sources can be an overwhelming task for pregnant women, considering the amount of information available, not only from web-based search engines, but also social media and society’s often unsolicited advice for pregnant women [30]. This can be difficult to filter, especially for women, who are new to this topic, with limited health literacy or nutrition competence. While the relatively high educational status of the study participants might go along with an increased health literacy [2], introducing a possible bias, the strengths of this study are the perspectives on a variety of different dietary forms, from omnivore to vegan, as well as the age range in the sample, from 23 to 38 year old pregnant women. Additionally, we received insights from women in all possible care models, from prenatal care exclusively by an obstetrician or a midwife to an alternating care model between the two professions. In future research, the results of this qualitative study could be extended with the usage of quantitative tools, such as the Patient Benefit Index (PBI) [45], assessing individual treatment goals and their subsequent achievement.

## Conclusion

This study identified potential for the improvement of German prenatal care in terms of nutrition guidance in pregnancy. This includes the implementation of personalised nutrition consultation in prenatal care, considering women’s individual needs and dietary forms as well as their coping abilities regarding health-related risks through foodborne illnesses and the amount of information they receive at the beginning of pregnancy. The usage of internet sources was shown to exacerbate already existing fears and anxiety in pregnant women, which was proposed to be effectively counteracted by adequate, empathetic and individualised guidance by healthcare professionals. Women in alternating prenatal care models between midwife and obstetrician were found to be more content with their overall nutrition consultation, especially compared to those solely cared for by an obstetrician. Insufficient and inconsistent nutrition information from healthcare professionals could be counteracted by implementing this topic in their professional education. As awareness and knowledge of pregnant women are essential in reducing the risks for APOs associated with poor nutrition, the communication on part of the healthcare professionals should reinforce the importance of nutrition in pregnancy.

### Electronic supplementary material

Below is the link to the electronic supplementary material.

Supplementary Material 1

## Data Availability

The datasets used and analysed during the current study are available from the corresponding author on request.

## Abbreviations

APO: Adverse pregnancy outcome
LBW: Low birth weight
PTB: Preterm birth
GDM: Gestational diabetes mellitus
LGA: Large for gestational age
SGA: Small for gestational age
BMI: Body mass index
LPEK: Local psychological ethics committee of the UKE
OSF: Open science framework
UKE: University Medical Center Hamburg-Eppendorf
SD: Standard deviation
PBI: Patient Benefit Index
