# Supplementary material for: Assessment of women’s needs, wishes and preferences regarding interprofessional guidance on nutrition in pregnancy – a qualitative study
Source: BMC Pregnancy Childbirth. 2024 Feb 21;24:154. doi: 10.1186/s12884-024-06351-z (PMC10880225; doi:10.1186/s12884-024-06351-z)
Supplement: Supplementary file 1 — Supplementary Material 1 [file 12884_2024_6351_MOESM1_ESM.docx]

**Supplement S1:** Focus group guide.

**Focus Group Guide**

Thank you very much for agreeing to participate in this study.

With this study we want to find out, what needs and wishes women have during pregnancy regarding the education and consultation on nutrition and oral health. For this, we want to imagine a “make-a-wish”-world together with you, meaning you can say anything that comes to mind and that you wish for, regardless of whether that service is currently offered or not.

As the education of midwives in Germany is currently changing and there is a lot of thought about possible interprofessional collaboration between different professions, the wishes you express can help to better align the care that is currently offered, with the actual needs of pregnant women.

Nutrition and oral health are important topics in pregnancy and can have an impact on how your pregnancy progresses, for example, the composition of the bacteria in your mouth may change, or you might have bleeding gums. That’s why it’s important to create an education about these relevant topics that fits the actual needs of pregnant women as optimally as possible.

To gain a comprehensive insight, we would like to look at how you have experienced education and consultation on nutrition and oral health so far, who has provided you with information and in what form, and anything else you would wish for beyond that.

We look forward to your personal opinion – there is no right or wrong. Our goal is to learn about your needs and wishes – these can be very individual. We also hope to have a pleasant conversation and a lively exchange during this session. You are welcome to comment or expand on anything another person says.

I will ask different questions and may steer the discussion in certain directions, but will try to keep my own speaking time as small as possible so that we can learn more from you.

I will record our session, which will be transcribed afterwards. This transcript will be anonymized, so that no conclusions can be drawn about your identity.

Do you have any questions?

Then I will start the recording now and we will begin with a brief introduction round.

| Main Questions | Follow-up Questions |
| --- | --- |
| Introductory question | |
| First, I would like to get an overview and ask, what kind of care model you have in your current pregnancy? | Prenatal care shared between midwife and obstetrician or only obstetrician?  Other form of prenatal care? |
| Specific topics: Nutrition | |
| To split our two topics today, I would like to begin with the topic of nutrition. This is not only about what you can and cannot eat during pregnancy, but also about your overall dietary habits. | |
| To what extent have you received counselling or education regarding nutrition during pregnancy and from whom?  Which topics were discussed?  If you did not receive education, what would you have wished for in that regard? | Potentially mention specific professions:   - Midwife - Obstetrician - Dietician - Family physician - Dentist |
| How did you experience this education?  What do you think about the handling of the topic of nutrition during pregnancy? | How positive/negative did you feel about the communication with you?  To what extent did you feel that your wishes were taken into account and taken seriously?  In what form were the information conveyed?  (in conversation, brochures, internet links, childbirth preparation courses) |
| To what extent did you feel that the education was sufficient?  What other resources or offerings would you have wished for? | How important is it for you to be advised on certain forms of diet?  At what point during pregnancy would you like to receive information? |
| Which nutrition-related topics or information are particularly important to you?  Which information on nutrition-related topics have been particularly helpful to you?  Where would you like to have more information? | How important is nutrition during pregnancy to you?  To what extent have you already dealt with this topic before pregnancy?  To what extent has your awareness about nutrition changed during pregnancy? |
| In times of the internet, we often obtain information through Google or social media, where it can sometimes be difficult to determine how reliable certain sources are. The sheer amount of information can also be overwhelming. | |
| How do you feel about this/experience this?  What topics do you research on the internet? | How do you deal with that? What helps you cope with the amount of information?  How do you find the information you need?  What (official) sources do you use?  What would help you filter information from the internet by relevance or credibility?  To what extent do you obtain information from your personal surroundings (family, friends)? |
| How do you make decisions regarding your nutrition during pregnancy?  To what extent does you knowledge about nutrition influence your decision on what to eat? | What is important to you when making decisions about your nutrition during pregnancy?  What influences your decision?   - Health of the baby/own health - Keeping your figure/avoiding too much weight gain - Food cravings - Gut feeling   Where do you wish for more/a different kind of support when making decisions or planning your nutrition? |
| Specific topics: Oral and dental health | |
| During pregnancy, oral health can change, for example due to hormonal changes or changes in dietary habits, which can lead to gum bleeding, for instance. Therefore, I would like to learn more about your experience with counselling and education regarding oral and dental health during your pregnancy. | |
| Introductory question: To what extent is the topic of oral and dental health during pregnancy of relevance to you? How important is this topic for you? | How do you feel about how the topic of oral and dental health is addressed during pregnancy? |
| How much consultation or education have you received on the topic of oral and dental health during your pregnancy?  What would you have wished for in this regard?  From whom did you receive information?  Did you find the education to be sufficient? | Potentially mention specific professions:   - Midwife - Obstetrician - Dietician - Family physician - Dentist   Did you bring up the topic yourself and what was the cause? |
| To what extent did you experience changes in your own oral and dental health during your pregnancy? | When would you decide to seek consultation?  Who would you prefer to receive help from?  What would contribute to relieving the decision-making process of seeking consultation? |
| Which information is particularly important to you in the education?  In what form would you like to receive this information?  How could preventive measures for oral health be integrated during pregnancy? | Information on which topics have been particularly helpful to you?  Where and on what would you wish for more information?  At what point during pregnancy would you have liked to receive education? |
| Interprofessional collaboration on both topics | |
| Finally, I would like to discuss with you how you felt overall about the care and education provided by all professional groups regarding both topics. | |
| To what extent and by whom were options for education discussed with you?  To what extent do you feel that you had sufficient time and opportunity to ask everything you wanted to discuss? | How important would it be for you to be referred to other professional groups? |
| How important would a collaboration between the different professional groups with whom you are in contact with during pregnancy be to you? | Who should be responsible for providing information on nutrition and oral health?  How important is it for you to have a clear responsibility or contact person?  In what setting would it be best for you to receive consultation on these topics?  How important is it for you to be cared for by one or more professional groups throughout the entire pregnancy? |
| To what extent do you feel that you have acquired competencies in these topics? | What in particular contributes to making you feel confident and competent?  Who has helped you with that? From whom would you like to receive help?  What would you need to feel more confident and competent? |
| At what point during pregnancy do you think an education on nutrition and oral health is the most sensible? | How have your questions and needs regarding these topics changed over time?  What information did you receive about these topics already before pregnancy?  What would help you feel well informed before and during pregnancy? |

**Supplement S2:** Interview guide.

**Interview Guide**

Thank you very much for agreeing to participate in this study.

With this study we want to find out, what needs and wishes women have during pregnancy regarding the education and consultation on nutrition and oral health. We have conducted several focus groups with pregnant women to gather insights, and now we would like to discuss your experiences as a healthcare provider in educating pregnant women.

Given that pregnant women receive care from various professionals during their pregnancy, interprofessional collaboration between different professions can be important. Your perspectives, along with the statements from pregnant women, can contribute to better aligning the provided care with the needs of pregnant women.

(Nutrition and oral health are crucial topics during pregnancy that can impact the course of pregnancy. Therefore, it is important to design education on these essential subjects to be optimally tailored to the actual needs of pregnant women.)

To gain a comprehensive understanding, we would like to explore how you structure counseling and education (on nutrition and oral health), what information you convey to pregnant women, and what you consider particularly important. Additionally, we are interested in hearing about your vision for an optimal educational or counseling situation and what could contribute to an improved integration of oral health into prenatal care.

We look forward to hearing your personal opinion; there is no right or wrong. Our goal is to understand your professional perspective. I will ask various questions and may guide the conversation in certain directions, but I will try to keep my own speaking time to a minimum so that we can learn more from you.

I will record our session, which will be transcribed afterwards. This transcript will be anonymized, so that no conclusions can be drawn about your identity.

Do you have any questions?

Then I will start the recording now and we will begin with a brief introduction round.

| Main Questions | Follow-up Questions |
| --- | --- |
| Introductory question | |
| Firstly, I would like to learn from you as an introduction, what profession you are engaged in and to what extent you work with pregnant individuals. | Prenatal care?  In which setting do you work? (clinical, non-clinical, employed, self-employed) |
| Specific topics: Nutrition | |
| To divide our topics a bit today, I would like to start with the topic of nutrition. This is not only about what is allowed and what is not during pregnancy but also about overall dietary behavior and how you experience the topic from your professional perspective. | |
| How do you educate women during their pregnancy on the topic of nutrition?  What content do you discuss? | In what form do you convey the information?  (through conversation, brochures, internet links) |
| How do you experience providing education to pregnant women?  How do you feel the topic of nutrition during pregnancy is generally addressed? | Is there a specific need for discussion or information?  If so, on which topics?  To what extent do you observe a changed awareness and behavior regarding nutrition among pregnant women? |
| Which specific topics or information are particularly important to you?  At what point during pregnancy do you consider education to be most fitting? | Where do you see a specific need for education?  How do you provide guidance on specific dietary patterns? |
| To what extent do you see nutrition education during pregnancy as the responsibility of your field?  What kind of support can your field provide in terms of education? | Do you experience inquiries from pregnant women?  How do you advise in such cases?  Do you refer to other professional groups, and if so, which ones?  In your opinion, which profession holds primary responsibility in this regard? |
| To what extent were you prepared for the topic of nutrition through your professional training? | How do you assess your own level of knowledge?  What knowledge gaps do you see in yourself or your professional group?  To what extent do you perceive the topic as adequately or inadequately covered in your professional training? |
| What factors or conditions facilitate or hinder you in educating pregnant women on the topic of nutrition? | What would assist you in educating pregnant women during prenatal care?  Are there specific conditions you would like to mention, and if so, what are they?  What sources are available to support you in counseling and education? |
| Specific topics: Oral and dental health | |
| During pregnancy, oral health can change, for example due to hormonal changes or changes in dietary habits, which can lead to gum bleeding, for instance. Therefore, I would like to learn more about how you manage counseling and education regarding oral health and dental care during pregnancy. | |
| Introductory question: To what extent is the topic of oral health and dental care during pregnancy significant for you? How relevant is this issue in your profession? | How do you feel the topic of oral health and dental care during pregnancy is generally addressed?  Do pregnant individuals approach you with this topic? |
| How do you educate women during their pregnancy on the topic of oral health and dental care?  What content do you discuss? | In what form do you convey the information?  (through conversation, brochures, internet links) |
| When do you perceive a need for education among pregnant women on this topic?  At what point during pregnancy do you consider education to be most fitting? | What motivates pregnant individuals to engage with this topic?  Do you observe specific needs or issues among pregnant women when it comes to dental care and oral health, and if so, what are they? |
| Which information is particularly important to you when educating about oral health and dental care during pregnancy? |  |
| To what extent do you see the responsibility of your field in educating about oral health and dental care during pregnancy? | What kind of support can your field provide in educating about oral health and dental care during pregnancy?  Do you refer to other professional groups, and if so, which ones?  In your opinion, which profession holds primary responsibility in this regard? |
| To what extent were you prepared for the topic of oral health during pregnancy through your professional training? | How do you assess your own level of knowledge?  What knowledge gaps do you see in yourself or your professional group?  To what extent do you perceive the topic as adequately or inadequately covered in your professional training? |
| What factors or conditions facilitate or hinder you in educating pregnant women about oral health and dental care?  How could oral health be integrated into prenatal care? | What would assist you in educating pregnant women during prenatal care?  Are there specific conditions you would like to mention, and if so, what are they?  What sources are available to support you in counseling and education?  What (financial) offerings should be available for pregnant women? |
| Interprofessional collaboration on both topics | |
| Lastly, I would like to discuss with you your experience with interprofessional collaboration. | |
| What advantages do you see in interprofessional collaboration, especially regarding education on nutrition and oral health during pregnancy?  What disadvantages do you see in interprofessional collaboration? | Who do you think should take on the task of educating about nutrition and oral health?  In which setting do you believe counseling would be best suited?  To what extent do you feel you have enough time and opportunity to discuss everything necessary with the patients? |
| Is there anything else you would like to share with us that has not been discussed in the conversation so far? | |

**Supplement S3:** Socio-demographic characteristics of focus group participants (n = 25).

| **Participant number** | **Age (years)** | **Number of children** | **Model of care** | **Diet** | **Week of pregnancy** |
| --- | --- | --- | --- | --- | --- |
| 01-01 | 26 | 0 | Obstetric | Omnivore | 38 |
| 01-02 | 30 | 0 | Obstetric | Vegetarian | 37 |
| 02-01 | 27 | 0 | Obstetric | Omnivore | 37 |
| 02-02 | 29 | 0 | Alternating | Omnivore, glutenfree | 39 |
| 03-01 | 31 | 0 | Midwifery | Omnivore | 20 |
| 03-02 | 34 | 1 (7 years old) | Midwifery | Vegetarian | 10 |
| 03-03 | 36 | 1 (1 year old) | Alternating | Omnivore | 31 |
| 03-04 | 32 | 1 (1 year old) | Obstetric | Omnivore | 31 |
| 03-05 | 33 | 0 | Obstetric | Omnivore | 20 |
| 03-06 | 30 | 0 | Obstetric | Omnivore | 34 |
| 04-01 | 35 | 0 | Alternating | Flexitarian | 35 |
| 05-01 | 32 | 0 | Midwifery | Pescetarian | 18 |
| 05-02 | 30 | 0 | Alternating | Omnivore | 29 |
| 05-03 | 26 | 0 | Alternating | Vegetarian, glutenfree | 38 |
| 05-04 | 38 | 1 (8 years old) | Alternating | Omnivore | 25 |
| 05-05 | 33 | 0 | Obstetric | Omnivore | 20 |
| 06-01 | 32 | 1 (3 years old) | Obstetric | Omnivore | 28 |
| 06-02 | 31 | 0 | Alternating | Vegetarian | 8 |
| 06-03 | 30 | 0 | Alternating | Omnivore | 23 |
| 06-04 | 23 | 0 | Obstetric | Pescetarian | 37 |
| 06-05 | 34 | 0 | Obstetric | Vegan | 40 |
| 07-01 | 34 | 0 | Alternating | Flexitarian | 27 |
| 07-02 | 28 | 1 (2 years old) | Midwifery | Vegan | 37 |
| 07-03 | 28 | 0 | Alternating | Vegetarian | 15 |
| 07-04 | 30 | 0 | Alternating | Omnivore | 28 |

**Supplement S4:** Developed category system reflecting the discussed topics in focus groups and interviews. Deductive: light grey background; inductive: white background.

| **Main categories** | **Subcategories** |
| --- | --- |
| Source of information | Experts/Health insurance app  Internet/Social media  Personal contacts |
| Topics | Food taboos/Risk of infection  Diet  Nutrient requirements  Intuitive eating  Health of unborn child  Pregnancy-related issues/Diseases:  GDM |
| Timing of consultation | |
| Communication between healthcare experts and pregnant women | Importance of communication  Communication with midwife  Communication with obstetrician |
| Offered services | Cost coverage  Offer for information/consultation |
| Professional education of experts | |
